# Comparative Metabolic and Stress-Related Responses to Adrenaline in Iberian and Landrace Pigs

**DOI:** 10.3390/ani16030354

**Published:** 2026-01-23

**Authors:** Manuel Lachica, Andreea Román, José Miguel Rodríguez-López, Lucrecia González-Valero, Consolación García-Contreras, Rosa Nieto, Ignacio Fernández-Fígares

**Affiliations:** 1 Departamento de Nutrición y Producción Animal Sostenible, Estación Experimental del Zaidín, CSIC, San Miguel 101, 18100 Armilla, Granada, Spain; manuel.lachica@eez.csic.es (M.L.); a.roman@eez.csic.es (A.R.); consolacion.garcia@eez.csic.es (C.G.-C.); rosa.nieto@eez.csic.es (R.N.); 2 IDEALISS, ULR 7519, UniLaSalle, Université d’Artois, 60000 Beauvais, France; jose.rodriguez@unilasalle.fr

**Keywords:** lipid metabolism, adrenergic stimulation, breed differences, fatty acid oxidation, glucose metabolism, glycolytic response, lipid mobilization, metabolic adaptation, muscle metabolism, stress physiology, pig

## Abstract

Differences in metabolic traits between traditional and modern pig breeds may influence their physiological responses to stress hormones. This study evaluated the in vivo metabolic effects of an acute adrenaline challenge in Iberian (obese, slow-growing) and Landrace (lean, fast-growing) pigs (*Sus scrofa domesticus*). Four Iberian and five Landrace barrows (≈50 kg body weight; BW) fitted with a carotid catheter received an injection of adrenaline (3 µg/kg BW), and serial blood samples were collected for 105 min. Adrenaline transiently increased plasma glucose (*p* < 0.001) and lactate (*p* < 0.001) concentrations, both peaking at 5 min post-injection. Iberian pigs showed higher plasma lactate (1.26 vs. 1.03 mM; *p* = 0.002), triglycerides (0.34 vs. 0.27 mM; *p* < 0.001), and non-esterified fatty acids (NEFA; 0.38 vs. 0.29 mM; *p* = 0.021), but lower glucose (4.80 vs. 5.03 mM; *p* = 0.010) than Landrace pigs, while cholesterol remained unaffected (*p* > 0.10). No breed × time interaction was detected for any metabolite. The relative increase in glucose reached +47% in Iberian and +27% in Landrace pigs, whereas lactate rose +140% and +113%, respectively, indicating stronger glycolytic activation in Iberian pigs. Despite the limited sample size, the results provide physiologically relevant evidence supporting increased metabolic flexibility in Iberian pigs, characterized by a heightened sensitivity to adrenergic stimulation and associated with enhanced lipolytic and glycolytic responses; however, these conclusions should be interpreted within the specific experimental conditions under which the study was conducted. These findings demonstrate that Iberian pigs have higher metabolic sensitivity to adrenergic stimulation, with enhanced lipolytic and glycolytic activity. In conclusion, breed-dependent differences in stress-related metabolism suggest that Iberian pigs are furnished with increased metabolic flexibility to face short-term stress.

## Simple Summary

Stress can strongly influence how farm animals use energy and nutrients. Adrenaline is a natural hormone released during stressful situations that helps animals prepare for “fight or flight” by changing how sugars and fats are used in the body. This study compared how two pig breeds with very different characteristics—the traditional, fatty Iberian pig and the modern, lean Landrace pig—respond to an injection of adrenaline. Blood samples taken over time revealed that adrenaline transiently elevated glucose and lactic acid concentrations in both breeds, although the magnitude of these changes was greater in Iberian pigs. Iberian pigs also had higher levels of fat-related substances in plasma, showing a greater release of energy from body fat. These results suggest that Iberian pigs are more sensitive to stress hormones, which may influence how their muscles and fat behave during stressful events. This could help explain differences in growth, fatness, and meat quality between traditional and modern pig breeds. Understanding these differences can help improve animal welfare and product quality in pig farming.

## 1. Introduction

Genetic selection for rapid growth rate, lean tissue deposition, and high feed efficiency has likely modified the release and/or action of hormones regulating nutrient utilization and storage and may have produced correlated changes in the ability of pigs to cope with immune or environmental challenges [1]. Among these hormones, catecholamines are particularly important because, together with cortisol, they mediate the animal’s physiological responses to stress and adaptation to novel environments. The sympathoadrenal system is also activated during physical exercise to enhance the supply of glucose and free fatty acids to active muscle. Moreover, catecholamines are involved in the metabolic preparation for the “fight or flight” response [2] and are considered the main hormones responsible for the temporary mobilization of energy stores through the breakdown of glycogen and triglycerides [3,4].

As a result, catecholamines profoundly affect carbohydrate, lipid, and protein metabolism. For instance, it is well established that swine mobilize fatty acids when catecholamines are injected [5]. Similarly, adrenaline increases blood glucose concentrations in exercising rats [6] and humans [7] and reduces circulating levels of branched-chain amino acids in humans, suggesting a decrease in muscle proteolysis [8].

The Iberian pig is a traditional breed native to the southwestern Iberian Peninsula, characterized by slow growth, low protein deposition capacity, and high carcass fatness [9]. It is also known for its high muscle protein turnover rate [10], enhanced protein degradation, and reduced muscle protein accretion compared with lean breeds, which together lead to reduced muscle protein accretion. Iberian pigs raised under the classical extensive system, *montanera*, endure variable food availability (acorns and grass) during their finishing phase. Despite the welfare benefits, managing these regime pigs could face nutritional stress (shortage) in contrast with pigs managed intensively with elevated growth pressure conditions, such as Landrace pigs. The main metabolic differences between Iberian and lean breeds are associated with the characteristic “thrifty metabolism” of Iberian pigs [11], reflecting long-term adaptation to the fluctuating nutritional and environmental conditions of traditional extensive production systems [12].

However, few studies have examined how these genotypic and metabolic differences influence carbohydrate, lipid, and protein metabolism under acute hormonal stimulation. Hormones may regulate the levels of metabolites that affect meat quality, therefore having an economic impact. To the best of our knowledge, no information is available regarding the effect of pig genotype on metabolic responses to an adrenaline challenge. Moreover, although high growth performance in lean breeds has been linked to greater stress and disease susceptibility [1], further experimental evidence is needed to support this hypothesis.

Therefore, we conducted an in vivo study in growing Iberian (fat, slow-growing) and Landrace (lean, fast-growing) pigs to question the hypothesis of differential metabolic responses to an adrenaline challenge. We aimed to assess systemic changes in circulating metabolites associated with energy homeostasis and to identify potential breed-related differences in the regulation of lipid and carbohydrate metabolism under adrenergic stimulation. Moreover, differential glycolytic and lipolytic responses may be modulated by the existing muscle-fiber composition and adipose tissue receptor density of each breed.

## 2. Materials and Methods

### 2.1. Animals and Experimental Design

Ten barrows—five Iberian (Silvela strain; Sánchez Romero Carvajal, Jabugo S.A., Cádiz, Spain) and five Landrace (Granja El Arenal, Córdoba, Spain)—with an average initial BW of 25 ± 0.4 kg, were used. Animals were individually housed in 2 m^2^ pens equipped with feeders and drinkers, in a controlled-environment room (21 ± 1.5 °C; 57% relative humidity). The photoperiod was fixed to 12 h of artificial light (08:00 to 20:00 h) and 12 h of darkness. All pigs had ad libitum access to water and were fed a standard barley–soybean meal diet formulated to contain 120 g crude protein/kg dry matter and 14.3 MJ metabolizable energy/kg dry matter.

The pigs were acclimated to their pens for four weeks before the start of the experiment. To minimize stress and avoid endogenous catecholamine release during sampling, animals were gently handled and accustomed to close human contact by daily petting throughout the acclimation period. The study was conducted in accordance with the European Union Directive 2010/63/EU on the protection of animals used for scientific purposes and approved by the Institutional Animal Care and Use Committee of the Estación Experimental del Zaidín (CSIC, Granada, Spain).

### 2.2. Surgical Procedures

To permit serial blood sampling and adrenaline injection without causing stress, each pig was surgically fitted with an indwelling carotid artery catheter, following the procedure described by Rodríguez-López et al. [13]. Briefly, pigs were fasted for 18 h prior to surgery. General anesthesia was induced by intramuscular administration of ketamine (15 mg/kg body weight (BW); Imalgene 1000, Merial, Barcelona, Spain) combined with azaperone (2 mg/kg BW; Stresnil, Esteve, Barcelona, Spain). Anesthesia was maintained throughout the surgical procedure using isoflurane (5% for induction and 0.5–2% for maintenance) delivered in oxygen (22–44 mL/kg BW/min) via a face mask. Analgesia and antispasmodic treatment consisted of a single intramuscular injection of 5 mL *N*-butyl hyoscine bromide plus sodium metamizol (Buscapina Compositum, Boehringer Ingelheim, Barcelona, Spain).

The surgical area was clipped, washed, and scrubbed three times with iodine soap, followed by the application of povidone iodine (7.5%) and 70% alcohol. All procedures were performed under strict aseptic and sterile conditions. The carotid artery was catheterized without occlusion, and the catheter was secured using a non-absorbable purse-string suture to maintain blood flow and minimize the risk of local infection. Catheter patency was confirmed daily by flushing 2 mL of physiological saline containing 5 IU heparin/mL. Catheters (Tygon; Cole-Parmer, Vernon Hills, IL, USA; internal diameter 1.02 mm, external diameter 1.78 mm, length 65 cm) were filled with physiological saline containing 250 IU heparin/mL (Fragmin, 5000 IU/0.2 mL; Pharmacia Spain S.A., Barcelona, Spain) and sealed.

Following surgery, pigs were transferred to individual metabolic cages. Feed intake was gradually restored, with animals receiving 25%, 60%, and 100% of their preoperative intake on days 1, 2, and 3 post-surgery, respectively. Rectal temperature remained within the normal range. A broad-spectrum antibiotic (Duphapen Strep; Fort Dodge Vet S.A., Girona, Spain) was administered intramuscularly for four days post-surgery (5–10 mg/kg BW/day). Skin sutures were removed approximately 10 days after surgery, at which time animals were considered fully recovered. During the 7 days following surgery, body temperature, feed, and water intake were monitored twice daily for all pigs. Normal feeding behavior and body temperature were observed for all pigs.

Catheter patency was maintained by daily flushing with sterile heparinized saline (250 IU heparin/mL in 0.9% NaCl; Pharmacia Spain S.A., Barcelona, Spain). A detailed description of catheter design, surgical procedures, and post-operative care has been published previously [13].

### 2.3. Adrenaline Challenge and Sampling

When pigs reached approximately 50 kg BW, they were fasted for 18 h and challenged with a single intracarotid injection of adrenaline (3 µg/kg BW; Adrenalina Braun 1 mg/mL, B. Braun Medical S.A., Rubí, Barcelona, Spain) diluted in 1 mL sterile saline and immediately followed by a 5 mL saline flush. Blood samples were collected from the carotid catheter at −15, 0, 5, 10, 15, 20, 25, 30, 45, 60, 75, 90, and 105 min relative to the injection in heparinized tubes (Monovette VetMed; Sarstedt, Nümbrecht, Germany) and kept on ice until centrifugation (4 °C, 1820× *g*, 30 min; Eppendorf 5810 R, Hamburg, Germany). Plasma was aliquoted and stored at −20 °C for subsequent analyses. One Iberian barrow was removed from the study because of procedural complications—a lack of patency in the implanted catheter precluded blood sampling.

### 2.4. Chemical Analyses

Plasma concentrations of glucose, lactate, triglycerides, cholesterol, and non-esterified fatty acids (NEFAs) were determined. Glucose, lactate, triglycerides, and cholesterol were measured colorimetrically using an automated BA 400 Biosystems analyzer (Biosystems S.A., Barcelona, Spain). NEFA concentrations were analyzed enzymatically using the acyl-CoA synthetase–acyl-CoA oxidase method according to the manufacturer’s protocol (FUJIFILM Wako Chemicals GmbH, Neuss, Germany). All metabolite assays were run in a single run except for NEFA. Intra-assay CVs were glucose 1.4%, lactate 1.0%, NEFA 4.7%, triglycerides 1.7%, and cholesterol 0.9%. Inter-assay CV of NEFA was 6.0%.

All analyses were performed at the Laboratorio de Técnicas Instrumentales, University of León (Spain) except for NEFA, carried out in the chemical laboratory of EEZ-CSIC.

### 2.5. Statistical Analysis

Each pig was considered the experimental unit. Data were analyzed using the MIXED procedure for repeated measures (SAS version 9.4; SAS Institute Inc., Cary, NC, USA). The statistical model included breed, sampling time, and their interaction (breed × time) as fixed effects. When significant effects were detected, mean comparisons were performed using Bonferroni’s post hoc test. Differences were considered statistically significant at *p* < 0.05. The statistical treatment of basal values was assessed by one-way analysis of variance using the GLM SAS procedure. A priori power analysis (α = 0.05, power = 0.80) indicated that a minimum of five experimental units per group was required to detect a difference of 105 µmolar, corresponding to an effect size of 1.8. The loss of one experimental unit represents a slight decrease in statistical power (1 − β = 0.77) from the nominal 0.8 power.

### 2.6. Availability of Data and Code

All data supporting the results of this study are available from the corresponding author upon reasonable request.

### 2.7. Disclosure of GenAI Use

Generative AI (OpenAI ChatGPT based on the GPT-4.1 architecture) was used exclusively for linguistic refinement and formatting of the manuscript text in accordance with the journal style. It was not used for data analysis, interpretation, or generation of scientific content.

## 3. Results

**Figure 1:**
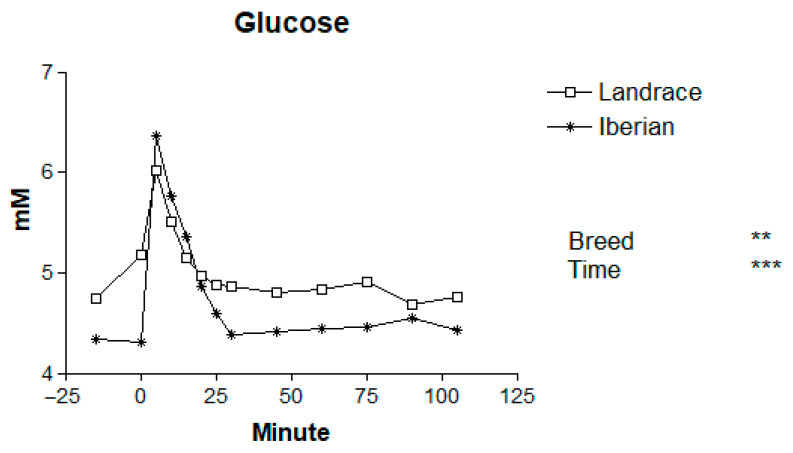
Responses to adrenaline of plasma glucose in Iberian (n = 4) and Landrace (n = 5) barrows. ** *p* < 0.01, *** *p* < 0.001.

**Figure 2:**
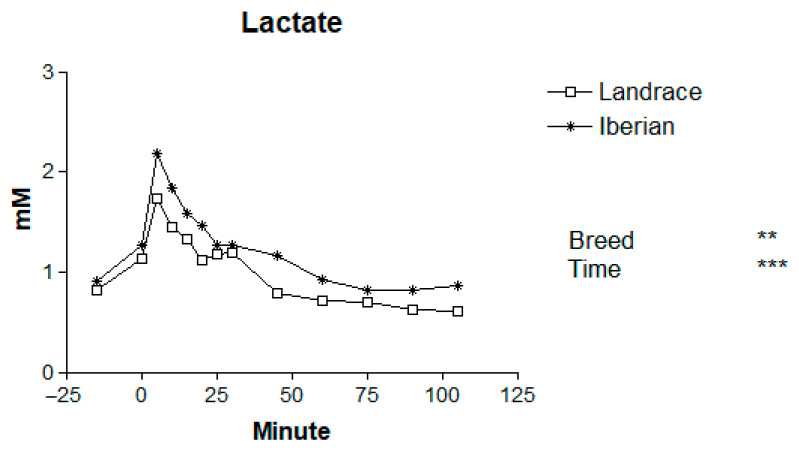
Responses to adrenaline of plasma lactate in Iberian (n = 4) and Landrace (n = 5) barrows. ** *p* < 0.01, *** *p* < 0.001.

**Figure 3:**
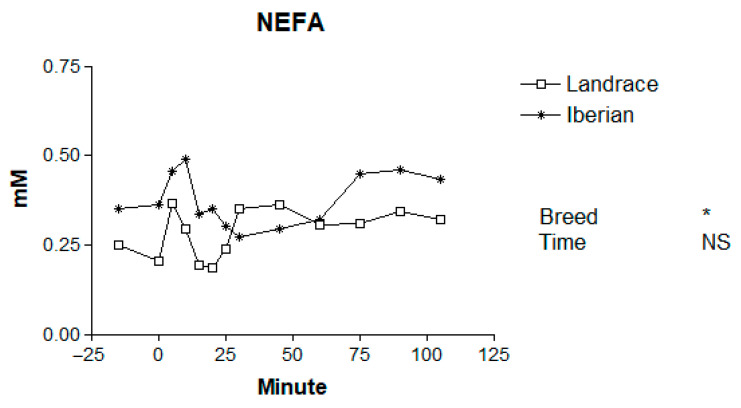
Responses to adrenaline of plasma non-esterified fatty acids (NEFAs) in Iberian (n = 4) and Landrace (n = 5) barrows. * *p* < 0.05, NS, not significant *p* > 0.10.

**Figure 4:**
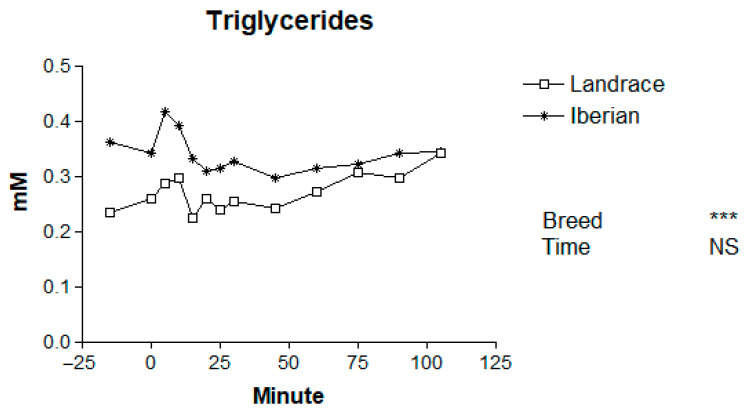
Responses to adrenaline of plasma triglycerides in Iberian (n = 4) and Landrace (n = 5) barrows. *** *p* < 0.001, NS, not significant *p* > 0.10.

**Figure 5:**
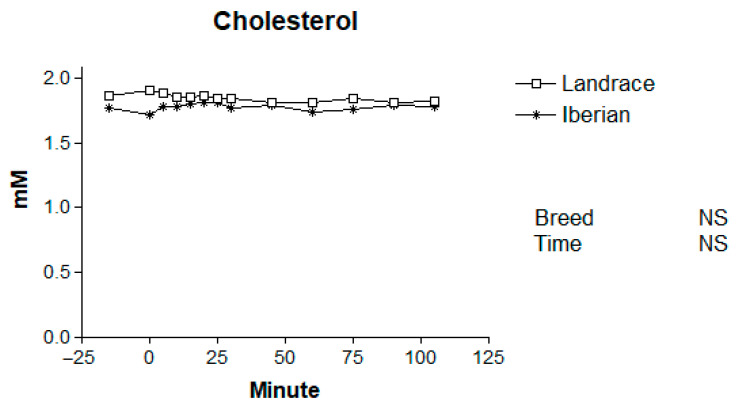
Responses to adrenaline of plasma cholesterol in Iberian (n = 4) and Landrace (n = 5) barrows. NS, not significant *p* > 0.10.

Basal levels of plasma metabolites for Iberian and Landrace pigs are shown in Table 1. The mean plasma concentrations of metabolites in Iberian and Landrace pigs following the adrenaline challenge are presented in Table 2, and the temporal patterns are illustrated in Figure 1, Figure 2, Figure 3, Figure 4 and Figure 5.

### 3.1. Plasma Glucose

Basal glucose levels were greater in Landrace compared with Iberian pigs (20.3%; *p* < 0.001; Table 2). Following the adrenaline injection, plasma glucose concentrations increased rapidly in both breeds (*p* < 0.001 for time effect; Figure 1). Maximum levels occurred at 5 min post-injection, representing a 36% increase relative to basal values. Glucose levels returned to baseline levels at 45 min in Landrace and at 30 min in Iberian pigs. The relative increment was greater in Iberian (47%) than in Landrace pigs (27%). Thereafter, glucose levels gradually returned to baseline by 45–60 min. Overall, Iberian pigs showed lower mean plasma glucose concentrations than Landrace pigs (*p* < 0.01), and no significant breed × time interaction was detected (*p* = 0.29). The absence of a significant breed × time interaction indicates that the overall temporal pattern of the metabolic response to adrenaline was similar in Iberian and Landrace pigs; that is, both breeds showed a rapid and transient change in plasma metabolites following the challenge, with peaks and subsequent return towards baseline occurring at comparable times. The area under the curve was greater in Landrace compared with Iberian pigs (869 vs. 756 mmol/120 min for Landrace and Iberian pigs, respectively. SEM = 19.4; *p* < 0.01).

### 3.2. Plasma Lactate

No differences in basal lactate levels were found between breeds (*p* > 0.10; Table 1). Adrenaline administration markedly increased plasma lactate concentration (*p* < 0.001 for time effect; Figure 2). The response peaked at 5 min post-injection, reaching 140% and 113% of basal levels for Iberian and Landrace pigs, respectively. Lactate levels returned to baseline levels at 45 min in Landrace and at 75 min in Iberian pigs. Mean lactate concentrations were significantly higher in Iberian than in Landrace pigs (*p* < 0.01), whereas the breed × time interaction was not significant (*p* = 0.996). However, no difference in the area under the curve for lactate was found between breeds (111 vs. 136 mmol/120 min for Landrace and Iberian pigs, respectively, SEM = 18.1; *p* > 0.10).

### 3.3. Plasma NEFA

No differences in basal NEFA levels were found between breeds (*p* > 0.10; Table 1). Plasma NEFA concentrations were higher in Iberian than in Landrace pigs (*p* = 0.021; Figure 3) following the adrenaline challenge. Although no time effect (*p* = 0.79) or breed × time interaction (*p* = 0.96) was observed, the overall NEFA response to adrenaline was transient, with a rapid increase shortly after injection followed by a progressive decline toward baseline values.

### 3.4. Plasma Triglycerides

Basal triglyceride levels were greater in Iberian compared with Landrace pigs (56.5%; *p* < 0.001; Table 1). Adrenaline treatment did not significantly affect plasma triglyceride concentrations over time (*p* = 0.32; Figure 4). However, Iberian pigs had higher mean triglyceride concentrations than Landrace pigs (*p* < 0.001). No significant breed × time interaction was detected (*p* = 0.83).

### 3.5. Plasma Cholesterol

No differences in basal cholesterol levels were found between breeds (*p* > 0.10; Table 1). Furthermore, no significant effects of breed, sampling time, or their interaction were detected for plasma cholesterol (*p* > 0.10; Figure 5). Mean cholesterol concentrations remained stable throughout the sampling period.

## 4. Discussion

Catecholamines, such as adrenaline and noradrenaline, are key mediators in the physiological preparation for acute stress, contributing to the classic “fight or flight” response by mobilizing energy substrates. The adrenal medulla releases adrenaline as an endocrine hormone that regulates metabolic processes and modulates behavior through interactions with the sympathetic nervous system and central noradrenergic pathways. In this study, we evaluated the metabolic effects of adrenaline in two pig breeds with contrasting growth potential and body composition: the lean, fast-growing Landrace and the fatty, slow-growing Iberian. Given their divergent genotype and metabolic phenotype, we hypothesized that they would exhibit distinct responses to acute adrenergic stimulation. The results confirmed this expectation, showing breed-dependent differences in plasma glucose, lactate, NEFA, and triglyceride dynamics following adrenaline injection.

### 4.1. Effect of Adrenaline on Plasma Glucose

In humans, adrenaline increases blood glucose by up to 50% above basal levels, followed by a return to baseline within approximately 30 min [14,15]. The transient rise in glucose observed in both breeds aligns with this pattern, although the magnitude of response differed: Iberian displayed a larger relative increase but maintained lower overall glucose concentrations than Landrace pigs. This breed effect reflects differences in carbohydrate metabolism. Adrenaline promotes hepatic and muscular glycogenolysis, as well as hepatic gluconeogenesis. The higher plasma glucose concentration in Landrace pigs agrees with the greater proportion of fast-twitch muscle fibers characteristic of this breed [16], which show enhanced glycogenolytic responsiveness to adrenergic stimulation, as reported in rats [17,18]. In contrast, hepatocyte studies revealed similar glycogen degradation capacity between Iberian and Landrace pigs, but higher gluconeogenic potential in Iberian pigs [19]. Elevated plasma lactate concentrations in Iberian pigs support increased glycolytic flux and possible stimulation of hepatic gluconeogenesis. Thus, while adrenaline may promote greater glycogenolysis in Landrace muscle, the higher lactate in Iberian pigs likely indicates a shift toward greater peripheral glycolytic activity and enhanced recycling of carbon substrates via gluconeogenesis.

### 4.2. Effect of Adrenaline on Plasma Lactate

The increase in plasma lactate following adrenaline injection reflects accelerated glycolysis and lactate release from skeletal muscle [20]. Iberian pigs showed a higher lactate response despite having less muscle mass than Landrace pigs, consistent with previous findings in other species where less muscular animals exhibit higher lactate responsiveness to adrenaline [21,22]. These differences may be linked to muscle fiber composition. Iberian pigs possess a higher proportion of type I (slow-oxidative) fibers and fewer type IIX (fast-glycolytic) fibers than Landrace pigs [16]. In rat and human skeletal muscle, slow-twitch oxidative fibers exhibit a higher density of β-adrenoceptors than fast-twitch glycolytic fibers, although the functional significance of this difference remains unclear and may be related to carbohydrate metabolism [23,24]. Data on adrenoceptor distribution among muscle fiber types in pigs are limited; however, assuming a similar pattern to that observed in rodents and humans, a higher abundance of β-adrenoceptors would be expected in Iberian compared with Landrace pigs. This assumption is supported by evidence showing greater adrenergic responsiveness in oxidative rat muscles [17]. Therefore, the greater lactate response of Iberian pigs likely results from higher β_2_-adrenoreceptor density and enhanced adrenergic sensitivity in oxidative fibers, overriding the larger glycogenolytic capacity of the fast-twitch muscle typical of Landrace pigs [25]. Under stress conditions, this heightened responsiveness could accelerate muscle glycogen depletion in Iberian pigs, increasing the likelihood of insufficient postmortem acidification and thus a higher incidence of dark, firm, and dry (DFD) meat if stress conditions were extended before animal slaughter. Indeed, DFD defects associated with inadequate pH decline at slaughter have been reported as a major quality issue in Iberian pigs [26].

Interestingly, diet characteristics and exercise did not affect muscle glycogen depots and their mobilization toward the formation of lactic acid via glycogenolysis and anaerobic glycolysis pathways in Iberian pigs at the finishing phase [26]. Although the present study did not directly assess carcass quality, the observed lactate response suggests possible implications for postmortem muscle metabolism and meat quality of major importance for the commercialization of Iberian products.

### 4.3. Effect of Adrenaline on Plasma NEFA and Triglycerides

White adipose tissue stores excess energy as triglycerides and releases NEFA through lipolysis when energy demand increases. Adrenaline activates this process via hormone-sensitive lipase and β-adrenergic receptor signaling. In the present study, Iberian pigs exhibited greater plasma NEFA concentrations after adrenaline injection, suggesting enhanced lipolytic responsiveness compared with Landrace pigs. This is in agreement with the lower respiratory quotient (RQ = CO_2_ production/O_2_ consumption) of Iberian pigs compared to Landrace, indicating a preferential oxidation of lipids [27]. Furthermore, Iberian pigs have a higher intake capacity than Landrace [28], so an identical intake would produce a stronger catabolic effect in the Iberian. The lipolytic effect was short-lived, with NEFA levels peaking shortly after injection and then declining, consistent with rapid metabolic clearance. Growth hormone (GH) is released from the pituitary as a potent lipolytic agent [29]. However, previous studies found no difference in growth hormone (GH) levels between Iberian and Landrace pigs [30], though Landrace pigs displayed higher GH-releasing factor-induced GH secretion [13], consistent with their greater lean growth capacity. Treatment with exogenous GH has been shown to increase adipose sensitivity to adrenaline and elevate plasma NEFA and glycerol [31]. Therefore, the heightened NEFA response of Iberian pigs in this study appears unrelated to GH but rather to intrinsic metabolic differences in adipose tissue. The literature on basal NEFA concentrations in obese vs. lean pigs is inconsistent. Some studies reported no difference [32], whereas others observed lower fasting NEFA in obese genotypes [33,34]. In the present experiment, basal NEFA values were at the lower end of the physiological range, suggesting that animals were calm and habituated to handling, minimizing confounding stress effects.

Possible differences in lipoprotein lipase (LPL) activity could also explain breed variations. Although no studies have evaluated LPL activity in Iberian pigs, comparable expression of LPL has been reported in the adipose tissue of Meishan (obese) and Landrace (lean) pigs [35]. Assuming similar LPL activity in Iberian (obese) and Landrace (lean) pigs, increased NEFAs would be expected in the former, with a greater number of fat tissue depots. In humans, obesity is associated with reduced β_2_-adrenoreceptor density and diminished lipolytic response to catecholamines [36,37]. Thus, the enhanced NEFA release in Iberian pigs may represent a unique metabolic adaptation differing from human obesity. Indeed, the rapid mobilization of lipids in Iberian pigs may represent an adaptive response to nutritional fluctuations resulting from a long history of extensive rearing systems. Plasma triglyceride concentrations did not change over time but were consistently higher in Iberian than in Landrace pigs. This finding agrees with earlier observations that obese pig genotypes often show elevated fasting triglycerides [32,33,34]. The lack of time response may reflect the fasting conditions of the challenge, as no dietary triglyceride input was present. LPL cleaves fatty acids from the 1 and 3 positions of the triacylglycerol molecule, controlling triacylglycerol partitioning between adipose tissues and muscles, thereby affecting fat storage or providing energy in the form of fatty acids for muscle growth [38]. The higher triglyceride levels in Iberian pigs could indicate reduced uptake of circulating NEFA for re-esterification or diminished LPL activity at the tissue level. No differences were found in fasting plasma cholesterol, and triglyceride concentrations remained unchanged after the adrenaline challenge, suggesting that acute circulating triglyceride levels are primarily driven by lipid mobilization from body stores rather than by de novo lipogenesis. As all pigs were fasted overnight before the adrenaline challenge, the fasting likely contributed to the relatively stable time profiles observed for NEFA, triglycerides, and cholesterol. In the fasted state, basal lipolysis and hepatic handling of circulating lipids are already activated, so the additional, short-term adrenergic stimulus may primarily modulate the level of lipid mobilization between breeds rather than producing large, further time-dependent changes in these plasma lipid variables. The absence of a significant breed × time interaction indicates that the overall temporal pattern of the metabolic response to adrenaline was similar in Iberian and Landrace pigs, that is, both breeds showed a rapid and transient change in plasma glucose and lactate following the challenge, with peaks and subsequent return towards baseline occurring at comparable times. In parallel, the main effects of breed and time demonstrate that, despite sharing a common response profile, Iberian pigs consistently differed from Landrace pigs in the magnitude of glucose, lactate, NEFA, and triglyceride concentrations. Collectively, the adrenaline challenge induced robust and rapid but transient increases in plasma glucose and lactate, confirming activation of glycolytic pathways in both breeds. Biologically, this suggests that the adrenergic stimulus activates similar regulatory pathways and kinetics in both breeds, while pre-existing breed-related differences in basal metabolism and responsiveness are expressed as parallel, rather than diverging, trajectories over time. Iberian pigs exhibited consistently higher concentrations of lactate, NEFA, and triglycerides, along with lower glucose levels compared with Landrace pigs, indicating a greater metabolic sensitivity to adrenergic stimulation. No changes were observed for cholesterol. These results demonstrate clear breed-dependent differences in energy substrate mobilization following acute adrenergic activation. Our results support the hypothesis that breed-related differences in metabolic and adrenergic sensitivity underlie divergent responses to environmental and physiological stress. The increased glycolytic and lipolytic responsiveness of Iberian pigs may reflect adaptive traits derived from extensive rearing and selection for robustness rather than growth [11,12] and provide greater metabolic flexibility to Iberian pigs. Considering the greater sensitivity of Iberian pigs to stress, specific welfare-oriented management practices, such as minimizing handling and extending lairage time (up to 24 h) prior to slaughter, may be advisable. In contrast, the lower metabolic sensitivity observed in Landrace pigs may represent an adaptive advantage under intensive production systems.

However, the study was conducted under controlled experimental conditions with a small sample size, which may limit statistical power and generalizability. Given the relatively small sample size and inter-individual variability, the study may also have been underpowered to detect subtler deviations in time course between breeds, so minor differences in kinetics cannot be completely ruled out and should be addressed in future work with larger cohorts or more intensive sampling. Sampling was performed in fasted animals, precluding evaluation of postprandial responses. An additional limitation of the experimental design is the absence of a saline-infused control group, which would have allowed discrimination of the metabolic effects specifically attributable to adrenaline from those arising from other stress-related factors during the challenge. Further, post-mortem muscle analyses were not included, limiting our ability to directly relate metabolic responses to meat quality outcomes.

Future studies should directly test the proposed molecular mechanisms by quantifying β_2_-adrenergic receptor and lipoprotein lipase (LPL) gene expression in both skeletal muscle and adipose tissue of Iberian and Landrace pigs. In addition, experimental models of chronic stress would allow assessment of cumulative metabolic and physiological differences between breeds, thereby clarifying how sustained adrenergic activation influences energy metabolism, stress resilience, and production traits. Integrating gene expression, enzyme activity, and proteomics analyses in muscle and adipose tissue will be essential to deepen our mechanistic understanding of the interplay between genotype, metabolism, and animal welfare.

## 5. Conclusions

Iberian and Landrace pigs differ markedly in their metabolic responses to an acute adrenaline challenge. Iberian pigs showed higher plasma lactate, triglyceride, and non-esterified fatty acid levels but lower glucose than Landrace pigs, indicating greater glycolytic and lipolytic activation. These findings reveal a higher metabolic sensitivity of Iberian pigs to adrenergic stimulation, consistent with their thrifty phenotype. Overall, breed-related differences in stress metabolism may influence energy balance, animal welfare, and meat quality.

## Figures and Tables

**Table 1:** Basal plasma concentrations of metabolites in Iberian (n = 4) and Landrace (n = 5) barrows before the intraarterial adrenaline challenge.

	Breed			
Trait	Landrace	Iberian	SEM	*p*-Value
Glucose, mM	7.12	5.87	0.178	0.001
Lactate, mM	0.82	0.91	0.113	0.575
NEFA, mM	0.25	0.35	0.094	0.453
Triglycerides, mM	0.23	0.36	0.038	0.042
Cholesterol, mM	1.86	1.78	0.181	0.733

**Table 2:** Mean plasma concentrations of metabolites in Iberian (n = 4) and Landrace (n = 5) barrows after an intraarterial adrenaline challenge (3 µg/kg BW) ^1^.

	Breed			*p*-Value		
Trait	Landrace	Iberian	SEM	Breed (B)	Time (T)	B × T
Glucose, mM	5.03	4.80	0.057	0.010	0.001	0.293
Lactate, mM	1.03	1.26	0.054	0.002	0.001	0.996
NEFA, mM	0.29	0.38	0.028	0.021	0.794	0.956
Triglycerides, mM	0.27	0.34	0.010	0.001	0.319	0.827
Cholesterol, mM	1.85	1.78	0.050	0.306	0.999	0.999

^1^ Average of 12 measurements (0, 5, 10, 15, 20, 25, 30, 45, 60, 75, 90, and 105 min relative to the adrenaline injection) for all metabolites.

## Data Availability

The raw data supporting the conclusions of this article will be made available by the authors on request.

## Abbreviations

The following abbreviations are used in this manuscript:

BW	Body Weight
DFD	Dark, Firm and Dry
GH	Growth Hormone
i.m.	Intra muscular
LPL	Lipoprotein Lipase
NEFA	Non-Esterified Fatty Acids
